# Diffusion tensor image features predict *IDH* genotype in newly diagnosed WHO grade II/III gliomas

**DOI:** 10.1038/s41598-017-13679-4

**Published:** 2017-10-17

**Authors:** Paul Eichinger, Esther Alberts, Claire Delbridge, Stefano Trebeschi, Alexander Valentinitsch, Stefanie Bette, Thomas Huber, Jens Gempt, Bernhard Meyer, Juergen Schlegel, Claus Zimmer, Jan S. Kirschke, Bjoern H. Menze, Benedikt Wiestler

**Affiliations:** 1 Department of Neuroradiology, Klinikum rechts der Isar, TU München, Germany; 2 Department of Computer Science, TU München, Germany; 3 Department of Neuropathology, Klinikum rechts der Isar, TU München, Germany; 4 Department of Neurosurgery, Klinikum rechts der Isar, TU München, Germany; 5 Institute for Advanced Study, TU München, Germany

## Abstract

We hypothesized that machine learning analysis based on texture information from the preoperative MRI can predict *IDH* mutational status in newly diagnosed WHO grade II and III gliomas. This retrospective study included in total 79 consecutive patients with a newly diagnosed WHO grade II or III glioma. Local binary pattern texture features were generated from preoperative B0 and fractional anisotropy (FA) diffusion tensor imaging. Using a training set of 59 patients, a single hidden layer neural network was then trained on the texture features to predict *IDH* status. The model was validated based on the prediction accuracy calculated in a previously unseen set of 20 gliomas. Prediction accuracy of the generated model was 92% (54/59 cases; AUC = 0.921) in the training and 95% (19/20; AUC = 0.952) in the validation cohort. The ten most important features were comprised of tumor size and both B0 and FA texture information, underlining the joint contribution of imaging data to classification. Machine learning analysis of DTI texture information and tumor size reliably predicts *IDH* status in preoperative MRI of gliomas. Such information may increasingly support individualized surgical strategies, supplement pathological analysis and highlight the potential of radiogenomics.

## Introduction

Large-scale genome-wide studies have dramatically broadened our insight into the complex genomic underpinnings of gliomas^1–3^. Perhaps the most prominent example of newly identified drivers of gliomagenesis are point mutations in either the codon 132 of *isocitrate dehydrogenase 1* (*IDH1*) or infrequently codon 172 of *IDH2*, which are very common in WHO grade II and III gliomas (70–80%) and rare in primary *de novo* glioblastomas (WHO grade IV, <10%)^4^. Mutant *IDH* catalyzes the formation of the onco-metabolite 2-hydroxyglutarate (2HG), which is thought to mediate the oncogenic effects of *IDH* mutation^5^. *IDH* mutant tumors most probably arise from a distinct cell of origin^6^. Consequently, *IDH* mutant and wild type tumors have dramatically different clinical courses: *IDH* wild type tumors have a significantly shorter survival. *IDH* mutational status is in fact a stronger prognosticator than WHO grade (III vs. IV)^7^. Using an epigenome-wide approach, we were able to show that a molecular classification of anaplastic gliomas based on *IDH* mutation and combined deletion of the short arm of chromosome 1 and the long arm of chromosome 19 (1p/19q co-deletion) is superior to a reference histopathological classification based on the original fourth edition of the WHO classification^8–10^. Other studies further substantiated that so-called “lower-grade glioma” (LGG; WHO grade II and III) are indeed subdivided into distinct molecular entities through *IDH* and 1p/19q^3^. All this evidence has led to the recognition of *IDH* mutant and wild type gliomas as distinct disease entities, which is also recognized in the updated WHO classification^11^. Considering that all tumor cells in an *IDH* mutant tumor carry the *IDH* mutation, it is not surprising that therapies targeting mutant *IDH* have been developed, both in the form of vaccinations^12^ and small molecules which inhibit mutant *IDH* ^13^. Also, for WHO grade III and IV tumors, patients with *IDH* mutant tumors have been shown to benefit from extended resection (encompassing both enhancing and non-enhancing tumor) as opposed to those with an *IDH* wildtype tumor^14^. Thus, a preoperative method to assess *IDH* status bears the potential to meaningfully influence initial surgical strategy even before first pathological specimens are available.

Besides this clinical reasoning for a radiological diagnosis of *IDH* status, in general, interest in how genomic information can be non-invasively assessed from imaging phenotype data has rapidly grown^15,16^. Machine learning algorithms, which excel at learning (non-linear) classifiers in multi-dimensional data sets, have greatly benefitted this field. One major advantage of most modern machine learning techniques in comparison to traditional univariate analyses is their ability to analyze a high-dimensional data set without prior selection of “candidate features”, by automatically weighing each feature, and hence leveraging the full information contained in the data set. Traditional univariate methods on the other hand either require prior knowledge or multiple testing of all input variables.

We hypothesized that integrative analysis of multimodal MRI data (B0 and FA) using a modern texture quantization method and a neural network classifier would yield a reliable non-invasive prediction of *IDH* status in WHO grade II and III low grade gliomas (LGG).

## Results

### Patient characteristics

Patient characteristics are summarized in Table 1. The training and validation cohort did not differ with respect to these parameters. *IDH* wild type tumors were a bit more common in the validation cohort (30% vs. 23%), though this difference was not significant (Fisher’s exact test, p = 0.548). As in large registries^17^, the majority of tumors were histologically classified as astrocytomas, making up roughly 2/3 of cases in both the training and validation cohort, and patients with an *IDH* wild type tumor tended to be older (mean age 47 years) than patients with an *IDH* mutant tumor (mean age 39 years, Welch t test, p = 0.1). Neurosurgical resection was performed a few days after the preoperative MRI.

**Table 1:** Baseline patient characteristics.

	Training cohort (n = 59)	Validation cohort (n = 20)	p
**Median age (SD, 95% CI), years**	40 (13.8, 37.4–44.6)	42.5 (16.4, 35.3–50.7)	0.625
**Sex, n**			0.417
Male	35	12	
Female	24	8	
**WHO grade II, n (%)**	35 (59%)	11 (55%)	0.3051
**WHO grade III, n (%)**	24 (41%)	9 (45%)	
**Astrocytoma, n (%)**	39 (66%)	12 (60%)	0.543
**Oligoastrocytoma, n (%)**	11 (19%)	6 (30%)	
**Oligodendroglioma, n (%)**	9 (15%)	2 (10%)	
***IDH*** **status, n (%)**			0.548
Mutant	46 (77%)	14 (70%)	
Wild type	13 (23%)	6 (30%)	

### Radiogenomic analysis

**Figure 1:**
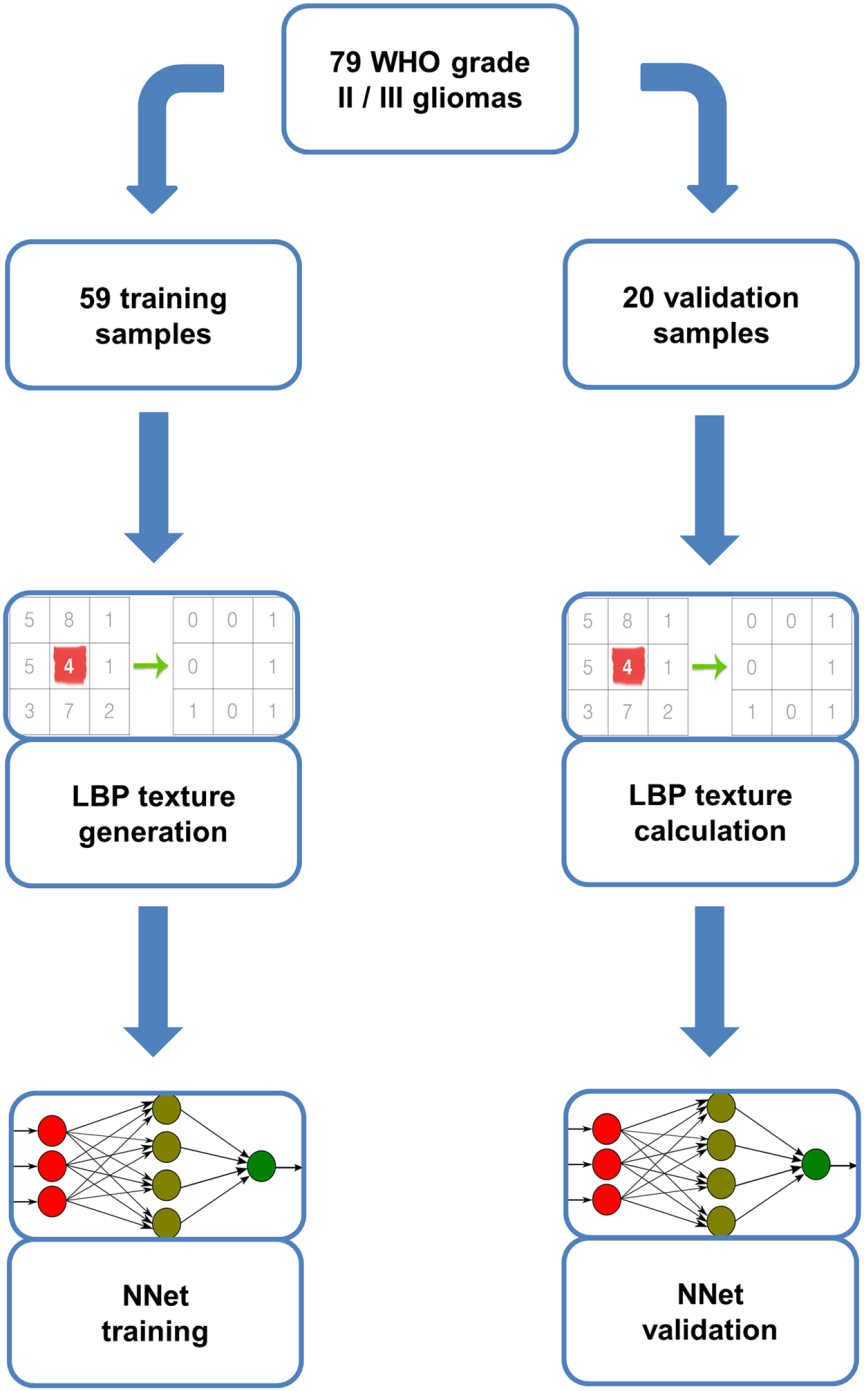
Overview of the image analysis pipeline.

**Figure 2:**
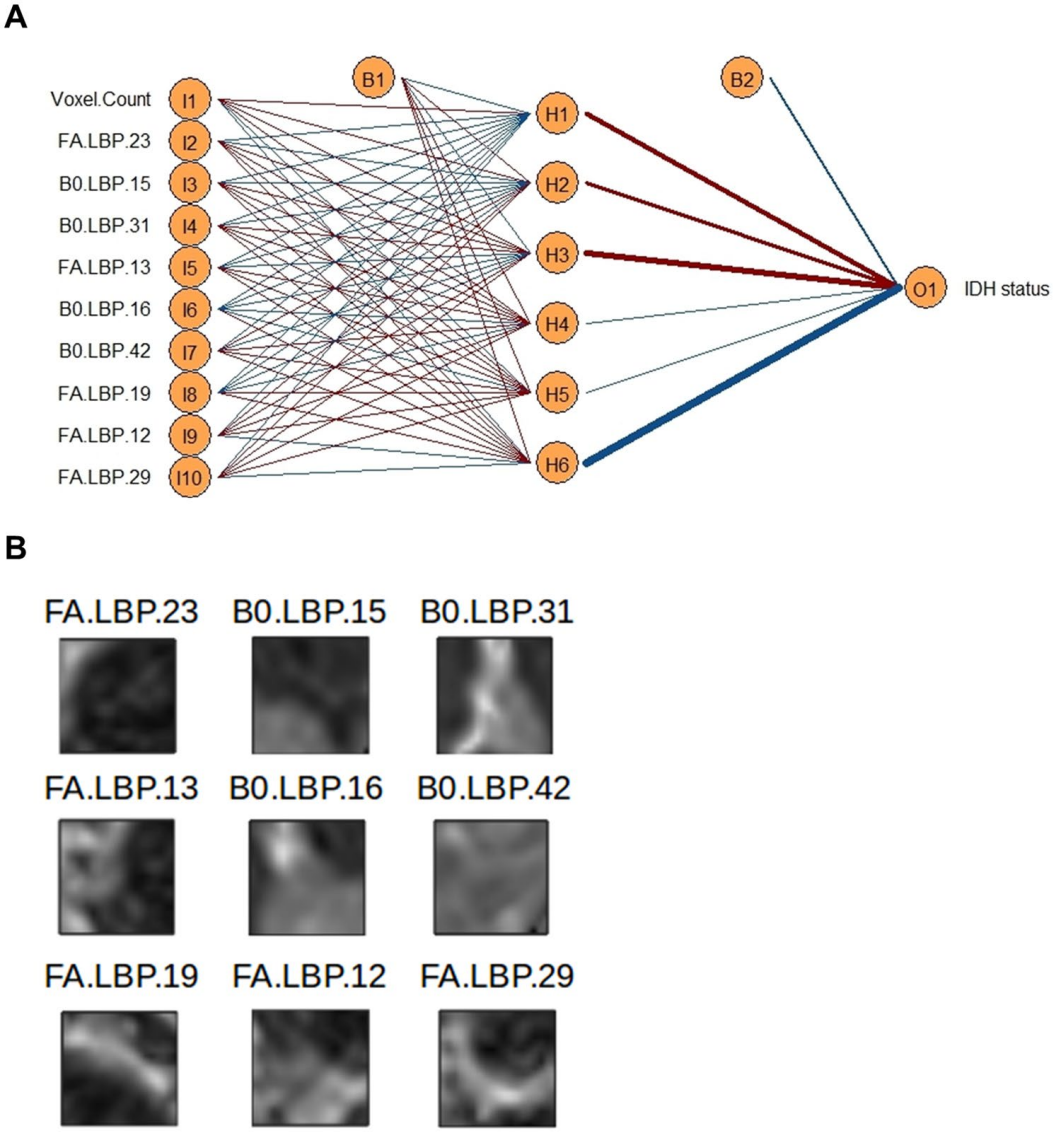
Plot of the final neural network classifier. (**A**) Please note that due to clarity, only the ten most important features (as per Garson importance) out of 101 are shown. Red lines indicate positive weights, blue lines negative weights. The thicker the line, the stronger the weight. (**B**) Representative examples (central two-dimensional slices of the respective cluster centers) of the 9 most important LBP textures. Please note that for illustrative purposes, only the central two-dimensional slice within each three-dimensional patch is shown for each texture. For the network analysis, three-dimensional textures have been used.

Using the pipeline shown in Fig. 1, we used the training cohort (59 patients) to generate 50 LBP texture features in the B0 and FA maps, respectively, as well as tumor size. These features were then used as input for a neural network classifier for *IDH* status. In the training cohort, this classifier (with six hidden units) reached an accuracy of 92% (54/59 cases; AUC = 0.921). While the neural network classifier integrates weighted information from all input features, we calculated relative feature importance using Garson’s algorithm to identify features contributing most to classification. Figure 2A shows a network plot of the ten most important features and Supplementary Table 1 lists all features and their respective importance. Furthermore, representative examples (central two-dimensional slices of the three-dimensional patches showing textures representing the learned texture patterns) of the 9 most important LBP textures are depicted in Fig. 2B.

**Figure 3:**
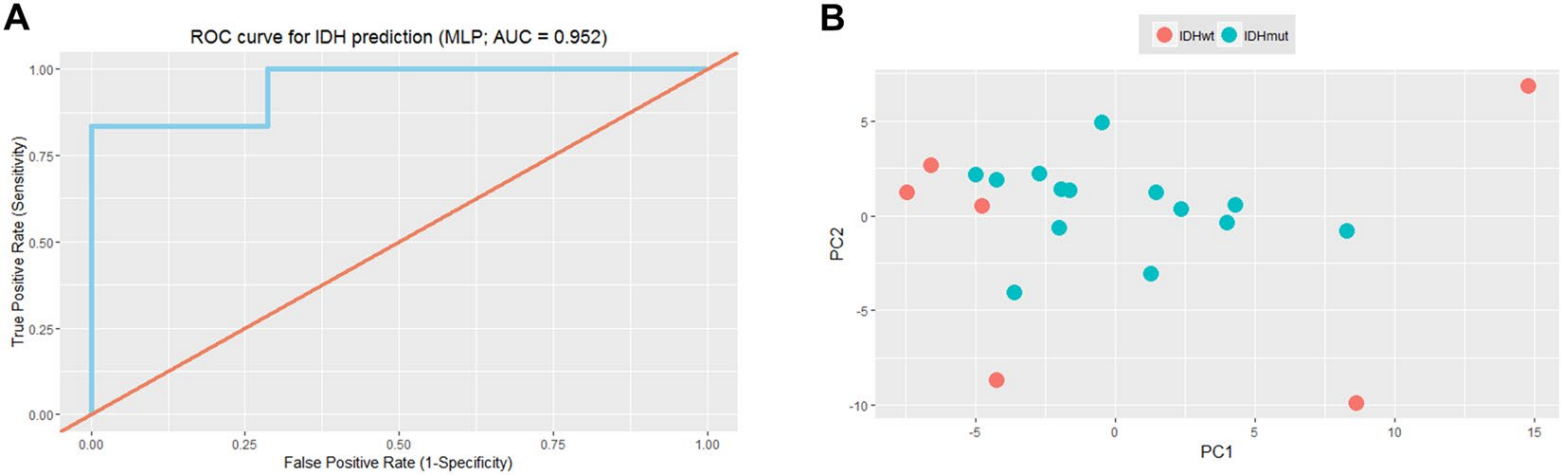
Validation cohort. (**A**) Receiver operating characteristic (ROC) curve for *IDH* status prediction in the 20 validation cases. Area under curve = 0.952. (**B**) PCA plot for the top 10 features in the network.

To validate the classifier, we calculated the texture features derived from the training cohort in an independent sample of 20 additional WHO grade II and III patients (details in Table 1). Here, the neural network classifier accurately predicted *IDH* status in 19/20 patients (95%) with an AUC of 95.2% (Fig. 3A). This accuracy was significantly higher than the “no information rate” (i.e. the percentage of correctly labelled samples if all samples were predicted to be *IDH* mutant; p = 0.007). We carefully inspected the misclassified *IDH* wild type tumor, but found no peculiarities. A principal component analysis (PCA) plot of the top 10 features in the test set showed a clustering of *IDH* mutant tumors, while *IDH* wild type were far more heterogeneously scattered, as suggested by the known higher genomic heterogeneity in *IDH* wild type tumors (Fig. 3B).

### Analyzing tumor size

**Figure 4:**
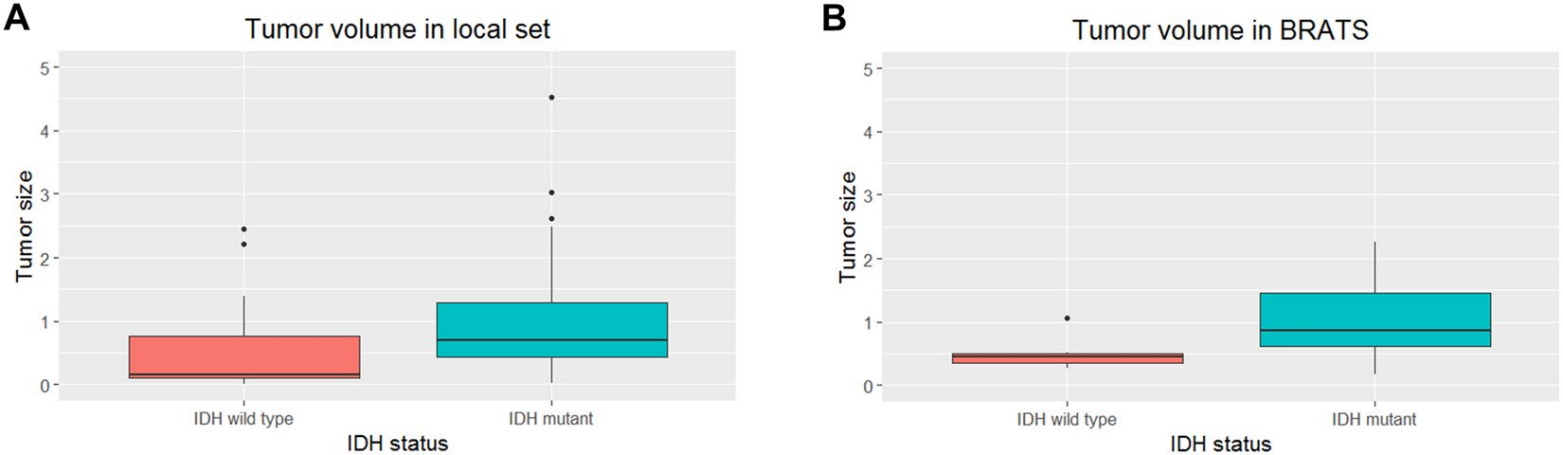
Tumor size of *IDH* mutant and wild type tumors. Boxplot of tumor size (separated by *IDH* status) in (**A**) our local data set and (**B**) the BRATS data set. Tumor size (voxel count) has been scaled to the mean of *IDH* mutant in each cohort.

Considering the importance of tumor size (voxel count) in the neural network, we found that in our cohort, *IDH* mutant tumors were significantly larger than their wild type counterparts (Wilcoxon rank sum test p = 0.008193; Fig. 4A). To validate this finding, we analyzed data from the 2015 brain tumor segmentation challenge (BRATS)^18^, which contains manually labelled whole tumor volumes for 44 WHO grade II and III gliomas as well as genotype information from the cancer genome archive (TCGA)^3^. In good agreement with our local data set, *IDH* wild type tumors in the BRATS set were also significantly smaller compared to *IDH* mutant tumors (Wilcoxon rank sum test p = 0.02715; Fig. 4B). Note that due to different segmentation strategies (B0 in our data set, T2/FLAIR in BRATS) and a smaller sample size in BRATS, data variability differs between BRATS and our local data set. However, a logistic regression model including only tumor size had a mediocre classification performance (AUC in training cohort = 0.727; AUC in validation cohort = 0.679), underlining the advantage of leveraging the full information contained in the data set through a neural network. Unfortunately, BRATS data do not contain DTI, precluding further validation of our classifier in this cohort.

## Discussion

In the present analysis, we have established a neural network classifier for prediction of *IDH* status from the preoperative MRI of WHO grade II and III glioma patients, relying on tumor volume and texture information from DTI data. This model reached accuracies of 92% in the training and 95% (19/20; AUC = 0.952) in the validation cohort.

In the current fourth edition of the WHO classification^8,11^, signs of anaplasia and mitotic activity distinguish WHO grade II and III gliomas. However, both criteria are subjective and prone to relevant inter-observer variability^19–21^. Furthermore, molecular subtypes associated with biology and prognosis have been identified^3,9,22^, and it was shown that outcome differences between WHO grade II and III gliomas rely far more on the distribution of these molecular subgroups than on true biological differences between WHO grade II and III gliomas^23^, suggesting that grade II and III gliomas may in fact be an entity better subdivided by molecular parameters. Consequently, we merged WHO grade II and III gliomas for our analysis. This is in accordance with large cohorts such as the cancer genome archive (TCGA), which also group WHO grade II and III gliomas as “lower grade glioma”.

Among molecular alterations in gliomas, *IDH* mutations are arguably the most prominent. Besides its diagnostic^11^ and prognostic value^7^, *IDH* status influences the clinical management of glioma patients and will do so even more in the future. In a large prospective cohort of 335 WHO grade III and IV gliomas, Beiko and colleagues demonstrated that *IDH* mutant tumors derive a clear overall survival benefit from total resection, including both enhancing and non-enhancing tumor, while *IDH* wild type tumors do not^14^. These results suggest individualized surgical strategies based on *IDH* status and therefore clearly necessitate diagnostic approaches for a preoperative *IDH* status assessment. To this end, several imaging-based methods have been developed, most prominently the 2HG magnetic resonance spectroscopy, which detects the onco-metabolite produced by mutant *IDH* ^24,25^. Although this technique promises a high diagnostic accuracy through the immediate detection of the metabolic activity of mutant *IDH*, it is still not in widespread use and the sequence as well as the post-processing routines need to be expertly set up at each center. On the other hand, our network classifier relies on a standard DTI sequence, which is readily available in most scanners, and publicly available classification algorithms. Furthermore, while the 2HG spectroscopy is naturally limited to detecting *IDH* mutations, machine-learning image analysis as presented here can possibly be extended to predict other relevant molecular targets as well, such as 1p/19q co-deletion or *MGMT* promoter methylation. In addition, molecular subgroups of gliomas, such as glioblastoma subgroups based on epigenome-wide methylation data^9,26^, could potentially be identified. Further, such techniques might be useful to help distinguish true tumor progression from treatment-associated pseudoprogression. Of note however, our classifier was not trained for these tasks and would need to be re-trained with appropriate samples to assess its performance and utility for these issues. While demonstrating a high diagnostic accuracy for predicting *IDH* mutations, our study thereby also highlights the potential of such approaches for radiogenomics in general.

Several imaging techniques better capturing underlying biology than traditional anatomical imaging (T1, T2, FLAIR) have recently been investigated for their ability to predict biology from the imaging phenotype. Diffusion tensor imaging (DTI) is an extension of diffusion weighted imaging (DWI). By measuring several gradient directions, DTI allows to calculate advanced scalars, most prominently fractional anisotropy (FA), which represents the directionality of the diffusion process. FA is thought to represent the cellular organization of tumors as well as their microenvironment, the extracellular matrix. In rat models, distinct patterns of diffusion directionality in the core and periphery of rat and human gliomas have been observed, which correlate well to histological findings in these models. Importantly, these patterns were not discernible in conventional MRI^27^. Consequently, FA values have been shown to indicate malignancy of gliomas, being associated with cell density and proliferation in human glioblastoma^28^ as well as WHO grade^29^. Furthermore, mean FA values in the contrast-enhancing tumor are significantly higher in glioblastoma compared to brain metastases, thereby facilitating the differentiation between these entities^30^. Recently, Xiong *et al*. reported that the maximum FA value in oligodendroglial tumors was significantly smaller in *IDH* mutant tumors compared to their wild type counterparts^31^. In addition to the FA maps, we also used the B0 images for texture analysis, as they are T2 weighted and in perfect co-registration with the FA maps. T2 texture information has been used to predict methylation status of the *O6-methylguanine-DNA methyltransferase* (*MGMT*) promoter in glioblastoma^32^ as well as the presence of a combined deletion of 1p and 19q in oligodendrogliomas^33^.

Among the ten features most important for *IDH* prediction was tumor volume. In our local data set, *IDH* mutant tumor were significantly larger (in terms of B0/T2 hyperintense whole tumor volume) than *IDH* wild type tumors. There have been few, but conflicting reports regarding differences in tumor size between tumors of different *IDH* status. Metellus *et al*. reported in WHO grade II gliomas that *IDH* wild type tumors were significantly larger on T2 images^34^. On the contrary, Lai *et al*. observed a larger size at diagnosis for *IDH* mutant tumors in a large cohort, albeit in glioblastoma^6^. In the BRATS data set, we could indeed confirm that at time of diagnosis, *IDH* mutant tumors are larger than their wild type counterparts. Importantly, time between preoperative MRI and tumor resection was short (a few days) in both groups in BRATS and our data set and therefore cannot explain this difference. Several factors might explain the difference in size at initial diagnosis between *IDH* mutant and wild type gliomas: First, *IDH* mutant tumors are known to predominantly occur in the frontal lobe^14^, allowing a tumor to grow larger before becoming symptomatic. Further, IDH mutant tumors grow slower than their wild type counter parts, giving the brain time to adjust and possibly delaying diagnosis. Importantly however, models solely relying on tumor size only had mediocre performance (AUC in training cohort = 0.727; AUC in validation cohort = 0.679), which was not significantly different from random labelling, clearly highlighting the necessity of analyzing the entire information contained in the data set. The remaining features were comprised of FA and B0 texture information, underlining the mutual contribution of texture information from both sequences for classification.

There are limitations to our study. As expected^4^, the majority of tumors (76%) carried an *IDH* mutation. To avoid missing *IDH* mutations, all tumors with a negative IDH1R132H immunohistochemistry were sequenced, thus a few samples without sufficient DNA for sequencing had to be excluded. Class imbalances can negatively impact a classifier. However, to account for this, the no information rate of our data set was used as a benchmark to validate model performance (i.e. if the model correctly predicts *IDH* status in more samples than expected just by chance). Furthermore, the data used for building the model was retrospectively collected in a single institution, containing 79 samples. The BRATS data unfortunately do not contain DTI data, precluding its use as a validation cohort for the classifier. However, we aimed to have a homogenous cohort of newly diagnosed patients with WHO grade II or III gliomas. In addition, this model was built only on B0 and FA information. How other modalities, especially perfusion and hypoxia imaging^35,36^, may meaningfully contribute to a predictive model for *IDH* status (or possibly other genomic aberrations) remains to be determined in future studies. Further, only six directions were acquired for tensor estimation. While this reduces scan time in clinical routine and can help to reduce motion artifacts, it remains unclear how a higher number of directions might influence texture generation and the classifier. However, an older study suggested that the influence of a higher number of directions is more pronounced for the eigenvalues than for tensor estimates (FA and mean diffusivity D)^37^. In addition, LBP textures rely on relative differences and not on absolute (B0/FA) values, suggesting that also for DTI with a higher number of directions, the presented classifier might be applicable (though it potentially might need to be retrained on the specific setup). Further, this classifier has been trained only on histologically proven WHO grade II/III gliomas. Its applicability to grade IV glioblastoma (which are mostly enhancing) is therefore unclear.

In summary, we present a machine learning classifier relying on preoperative DTI data to accurately predict *IDH* mutational status in newly diagnosed WHO grade II and III gliomas, highlighting the potential of non-invasive, MRI-based genotype assessment. Considering the clinical need for reliable determination of *IDH* mutational status and the emerging use of mutant *IDH* as a therapeutic target, such models have the potential not only to meaningfully complement pathological evaluation of the tumor but even to precede it and therefore support individualized surgical strategies.

## Materials and Methods

### Patients and MR imaging

This study was approved by the ethics committee of the TUM, and informed consent was waived for this retrospective analysis. In total, this study included 79 patients with a newly diagnosed WHO grade II or III glioma (astrocytoma, oligodendroglioma or mixed oligoastrocytoma) based on histopathological diagnosis, who had received a preoperative MRI including a standard 6 direction DTI (TR/TE 7665/85ms, spatial resolution 2 × 2 × 2mm³, b value 1000 s/mm²) on a 3 Tesla Philips Achieva between 2011 and 2015 and had *IDH* mutational status available. Patients without *IDH* status, preoperative DTI or severe motion artifacts were excluded. All remaining eligible patients, identified using our local pathology database, were included. Fractional anisotropy (FA) maps were automatically calculated from DTI raw data using software supplied by the MRI manufacturer. Briefly, gradient-direction images are co-registered on the B0 image, and a linear system is used to estimate the diffusion tensor. Patient demographics and histological diagnosis according to the fourth edition of the WHO classification^8^ were obtained from medical records. All methods were carried out in accordance with the relevant guidelines and regulations.

Additionally, 44 cancer imaging archive (TCIA; http://www.cancerimagingarchive.net/) WHO grade II and III samples from the brain tumor segmentation challenge (BRATS^18^; http://braintumorsegmentation.org) were collected (https://www.smir.ch/BRATS/Start2015) to serve as a validation cohort for tumor size differences based on *IDH* status. For these samples, manually segmented labels (on FLAIR and T2 images) were used to extract whole tumor volumes. In parallel, genotype data for these samples was compiled from The Cancer Genome Archive (TCGA; http://cancergenome.nih.gov/).

### Image analysis

B0 images were semi-automatically segmented using the publicly available software ITK-Snap (version 3.4.0), with a threshold-based approach^38^. Segmentation target was whole tumor, i.e. all areas of signal abnormality (including solid tumor, infiltrating tumor and edema) were included in a unified tumor mask. An example is shown in Supplementary Figure 1. In all cases, the resulting segmentation was visually inspected by an investigator blinded to *IDH* status (P.E., experienced neuroradiologist) and manually refined where necessary. To evaluate tumor segmentation, the neuroradiologist had access to all sequences, including FLAIR and contrast-enhanced T1. However, rigid registration (using the Elastix framework) of DTI and FLAIR revealed small but relevant registration differences (in terms of minimizing a mutual information – based cost function). Due to these reasons, we chose to perform segmentation of tumors on B0 images.

Consecutively, tumor texture was quantified using three-dimensional local binary patterns (LBP). LBPs are popular rotational invariant texture descriptors, which have recently been extended to three-dimensional representations^39^.

#### LBP feature calculation

For each voxel p in the segmented tumor, n neighborhood voxels in a sphere with radius r, centered at voxel p, are sampled. The intensity values of the neighborhood voxels are then compared to the intensity value of voxel p. That is, neighborhood voxels are set to 1 if they are more intense, and to 0 otherwise. This binary neighborhood representation is then approximated by a combination of spherical harmonics. More in particular, coefficients of spherical basis functions are computed such that the resulting function approximates the binary neighborhood representation. The LBP features are then given by the L2-norms of these coefficients and the kurtosis of the original intensities of the neighborhood voxels. In this study, LBP features were calculated for radii of 1 mm, 2 mm and 3 mm and concatenated into one single feature vector.

#### LBP clustering

During a training phase, the feature vectors were calculated for all voxels within the tumor regions of all the training patients. We then performed clustering of all the feature vectors using k-means clustering with k = 50 clusters. The centroids of these clusters are referred to as the learned texture patterns and represent distinctive patterns specific to our dataset.

To get a texture representation for the previously unseen validation cohort, raw LBP features are calculated for all voxels within the tumor mask, as previously described, and are then clustered towards the learned texture patterns. The final feature vector, describing the texture of the entire tumor, is then set to the relative occurrence of each of the learned texture patterns.

### Genotype analysis

All tumor samples were formalin fixed and paraffin embedded. 3 μm thick sections were cut, using a rotary microtome (HM355S, Thermo Fisher Scientific), mounted on SuperFrost Plus slides, and then stained with hematoxylin and eosin. Screening and grading was performed by two neuropathologists, assuring sections with representative tumor content were selected.

IDH1-R132H immunohistochemistry was performed on deparaffinized and rehydrated sections, using the Benchmark immunohistochemistry system (Ventana XT, Ventana Medical Systems, Tucson, AZ, USA). Slides were incubated for 31 min with mIDH1R132H hybridoma supernatant (clone H09, dilution 1:1, standard Cell Conditioner 1 M pretreatment and amplification). Cytoplasmic staining in tumor cells was considered positive.

Tumors showing no IDH1 immunoreactivity were then sequenced for *IDH1* and *IDH2* mutations. DNA was extracted from tumor tissue using the QIAmp DNA FFPE Tissue Kit (QIAGEN Venlo, Netherlands). PCR and sequencing was performed as previously described^40^.

### Pattern classification

The resulting 101 image features (voxel count of segmented area, frequencies of the 50 LBP textures in B0 and FA, respectively) were used as input variables in a single hidden layer neural network. Patients were randomly separated 3:1 into a training cohort (59 patients) and a validation cohort (20 patients). For activation, a sigmoid function was used, with a single logistic output neuron. The neural network was fitted using the R package “nnet”. The R package “caret” was used to perform a grid search over the tuning parameters “size” (number of hidden units) and “decay” (parameter for weight decay) on the 59 training cases optimizing for classification accuracy in the training cohort. We used Garson’s algorithm to calculate the relative importance of input variables in the final neural network^41^. Network visualization was performed with the “NeuralNetTools” package.

### Validation

The final model was applied to the 20 validation cases that had not been used during model generation. The model’s performance was assessed in this independent validation cohort calculating classification accuracy and the area under the curve (AUC) of the receiver operating characteristic (ROC) using the “caret” and “pROC” packages. We performed an exact binomial test, using the rate of *IDH* mutant tumors in the validation cohort (i.e. the “no information rate”) as probability of success, to assess whether the classification accuracy in the validation cohort is above chance.

Wilcoxon’s rank sum test was used to compare median tumor size between *IDH* mutant and wild type tumors. To calculate the individual performance of tumor size for *IDH* status prediction, we performed a univariate logistic regression and calculation of the AUC from the ROC curve. Fisher’s exact test was employed to compare distributions in a 2 × 2 contingency table. Student’s t test, assuming unequal variances between groups, was employed for continuous data. Analyses were carried out using R version 3.3. A p value < 0.05 was considered significant.

### Data Availability

The R scripts used to train the network classifier as well as methods for generating LBP textures from three-dimensional image data are available from the corresponding author (BW) upon request.

## Electronic supplementary material

Supplementary Files

## References

[1] Parsons DW (2008). An integrated genomic analysis of human glioblastoma multiforme. Science.

[2] Brennan CW (2013). The somatic genomic landscape of glioblastoma. Cell.

[3] Brat DJ (2015). Comprehensive, Integrative Genomic Analysis of Diffuse Lower-Grade Gliomas. N. Engl. J. Med..

[4] Yan H (2009). IDH1 and IDH2 mutations in gliomas. N. Engl. J. Med..

[5] Dang L (2009). Cancer-associated IDH1 mutations produce 2-hydroxyglutarate. Nature.

[6] Lai A (2011). Evidence for sequenced molecular evolution of IDH1 mutant glioblastoma from a distinct cell of origin. J. Clin. Oncol..

[7] Hartmann C (2010). Patients with IDH1 wild type anaplastic astrocytomas exhibit worse prognosis than IDH1-mutated glioblastomas, and IDH1 mutation status accounts for the unfavorable prognostic effect of higher age: implications for classification of gliomas. Acta Neuropathol..

[8] Louis DN (2007). The2007 WHO classification of tumours of the central nervous system. Acta Neuropathol..

[9] Wiestler B (2014). Integrated DNA methylation and copy-number profiling identify three clinically and biologically relevant groups of anaplastic glioma. Acta Neuropathol..

[10] Wiestler B (2013). ATRX loss refines the classification of anaplastic gliomas and identifies a subgroup of IDH mutant astrocytic tumors with better prognosis. Acta Neuropathol..

[11] Louis DN (2016). The2016 World Health Organization Classification of Tumors of the Central Nervous System: a summary. Acta Neuropathol..

[12] Schumacher T (2014). A vaccine targeting mutant IDH1 induces antitumour immunity. Nature.

[13] Rohle D (2013). An inhibitor of mutant IDH1 delays growth and promotes differentiation of glioma cells. Science.

[14] Beiko J (2014). IDH1 mutant malignant astrocytomas are more amenable to surgical resection and have a survival benefit associated with maximal surgical resection. Neuro. Oncol..

[15] Ellingson BM (2015). Radiogenomics and imaging phenotypes in glioblastoma: novel observations and correlation with molecular characteristics. Curr. Neurol. Neurosci. Rep..

[16] Gillies RJ, Kinahan PE, Hricak H (2016). Radiomics: Images Are More than Pictures, They Are Data. Radiology.

[17] Ostrom QT (2014). CBTRUS Statistical Report: Primary Brain and Central Nervous System Tumors Diagnosed in the United States in 2007-2011. Neuro. Oncol..

[18] Menze B (2015). The Multimodal Brain Tumor Image Segmentation Benchmark (BRATS). IEEE Trans. Med. Imaging.

[19] Kros JM (2011). Grading of gliomas: the road from eminence to evidence. J. Neuropathol. Exp. Neurol..

[20] Kros JM (2007). Panel review of anaplastic oligodendroglioma from European Organization For Research and Treatment of Cancer Trial 26951: assessment of consensus in diagnosis, influence of 1p/19q loss, and correlations with outcome. J. Neuropathol. Exp. Neurol..

[21] van den Bent MJ (2010). Interobserver variation of the histopathological diagnosis in clinical trials on glioma: a clinician’s perspective. Acta Neuropathol..

[22] Suzuki H (2015). Mutational landscape and clonal architecture in grade II and III gliomas. Nat. Genet..

[23] Reuss DE (2015). IDH mutant diffuse and anaplastic astrocytomas have similar age at presentation and little difference in survival: a grading problem for WHO. Acta Neuropathol..

[24] Choi C (2012). 2-hydroxyglutarate detection by magnetic resonance spectroscopy in IDH-mutated patients with gliomas. Nat. Med..

[25] Choi C (2016). Prospective Longitudinal Analysis of 2-Hydroxyglutarate Magnetic Resonance Spectroscopy Identifies Broad Clinical Utility for the Management of Patients With *IDH* -Mutant Glioma. J. Clin. Oncol..

[26] Sturm D (2012). Hotspot mutations in H3F3A and IDH1 define distinct epigenetic and biological subgroups of glioblastoma. Cancer Cell.

[27] Zhang J (2007). Unique patterns of diffusion directionality in rat brain tumors revealed by high-resolution diffusion tensor MRI. Magn. Reson. Med..

[28] Beppu T (2005). Fractional anisotropy value by diffusion tensor magnetic resonance imaging as a predictor of cell density and proliferation activity of glioblastomas. Surg. Neurol..

[29] Inoue T (2005). Diffusion tensor imaging for preoperative evaluation of tumor grade in gliomas. Clin. Neurol. Neurosurg..

[30] Wang S (2014). Diagnostic utility of diffusion tensor imaging in differentiating glioblastomas from brain metastases. Am. J. Neuroradiol..

[31] Xiong J (2016). Detecting isocitrate dehydrogenase gene mutations in oligodendroglial tumors using diffusion tensor imaging metrics and their correlations with proliferation and microvascular density. J. Magn. Reson. Imaging.

[32] Drabycz S (2010). An analysis of image texture, tumor location, and MGMT promoter methylation in glioblastoma using magnetic resonance imaging. Neuroimage.

[33] Brown R (2008). The Use of Magnetic Resonance Imaging to Noninvasively Detect Genetic Signatures in Oligodendroglioma. Clin. Cancer Res..

[34] Metellus P (2010). Absence of IDH mutation identifies a novel radiologic and molecular subtype of WHO grade II gliomas with dismal prognosis. Acta Neuropathol..

[35] Kickingereder P (2015). IDH mutation status is associated with a distinct hypoxia/angiogenesis transcriptome signature which is non-invasively predictable with rCBV imaging in human glioma. Sci. Rep..

[36] Wiestler B (2016). Multiparametric MRI-based differentiation of WHO grade II/III glioma and WHO grade IV glioblastoma. Sci. Rep..

[37] Ni H (2006). Effects of number of diffusion gradient directions on derived diffusion tensor imaging indices in human brain. Am. J. Neuroradiol..

[38] Yushkevich PA (2006). User-guided 3D active contour segmentation of anatomical structures: significantly improved efficiency and reliability. Neuroimage.

[39] Banerjee, J., Moelker, A., Niessen, W. J. & van Walsum, T. 3D LBP-Based Rotationally Invariant Region Description. *Comput. Vis. - ACCV 2012 Work. ACCV 2012 Int*. *Work. Daejeon, Korea, Novemb. 5–6, 2012, Revis. Sel. Pap*. Part I, 26–37 (2013).

[40] Hartmann C (2009). Type and frequency of IDH1 and IDH2 mutations are related to astrocytic and oligodendroglial differentiation and age: a study of 1,010 diffuse gliomas. Acta Neuropathol..

[41] Garson GD (1991). Interpreting neural-network connection weights. Artif. Intell. Expert.

