# Supplementary material for: Diffusion tensor image features predict IDH genotype in newly diagnosed WHO grade II/III gliomas
Source: Sci Rep. 2017 Oct 17;7:13396. doi: 10.1038/s41598-017-13679-4 (PMC5645407; doi:10.1038/s41598-017-13679-4)
Supplement: Supplementary file 1 — Supplementary Files [file 41598_2017_13679_MOESM1_ESM.pdf]

Diffusion tensor image features predict *IDH* genotype in newly diagnosed WHO grade II / III gliomas

Paul Eichinger, MD[1]*, Esther Alberts, MSc[1,2]*, Claire Delbridge, MD[3], Stefano Trebeschi, PhD[1], Alexander Valentinitsch, PhD[1], Stefanie Bette, MD[1], Thomas Huber, MD[1], Jens Gempt, MD[4], Bernhard Meyer, MD[4], Juergen Schlegel, MD[3], Claus Zimmer, MD[1], Jan S Kirschke, MD[1], Bjoern H Menze, PhD[2,5] and Benedikt Wiestler, MD[1]

[1]Department of Neuroradiology, Klinikum rechts der Isar, TU München, Germany
[2]Department of Computer Science, TU München, Germany
[3]Department of Neuropathology, Klinikum rechts der Isar, TU München, Germany
[4]Department of Neurosurgery, Klinikum rechts der Isar, TU München, Germany
[5]Institute for Advanced Study, TU München, Germany
* These authors contributed equally

Supplementary Figure 1

Example of a B0 (A) and FA (B) image, with the segmentation overlaid in red.

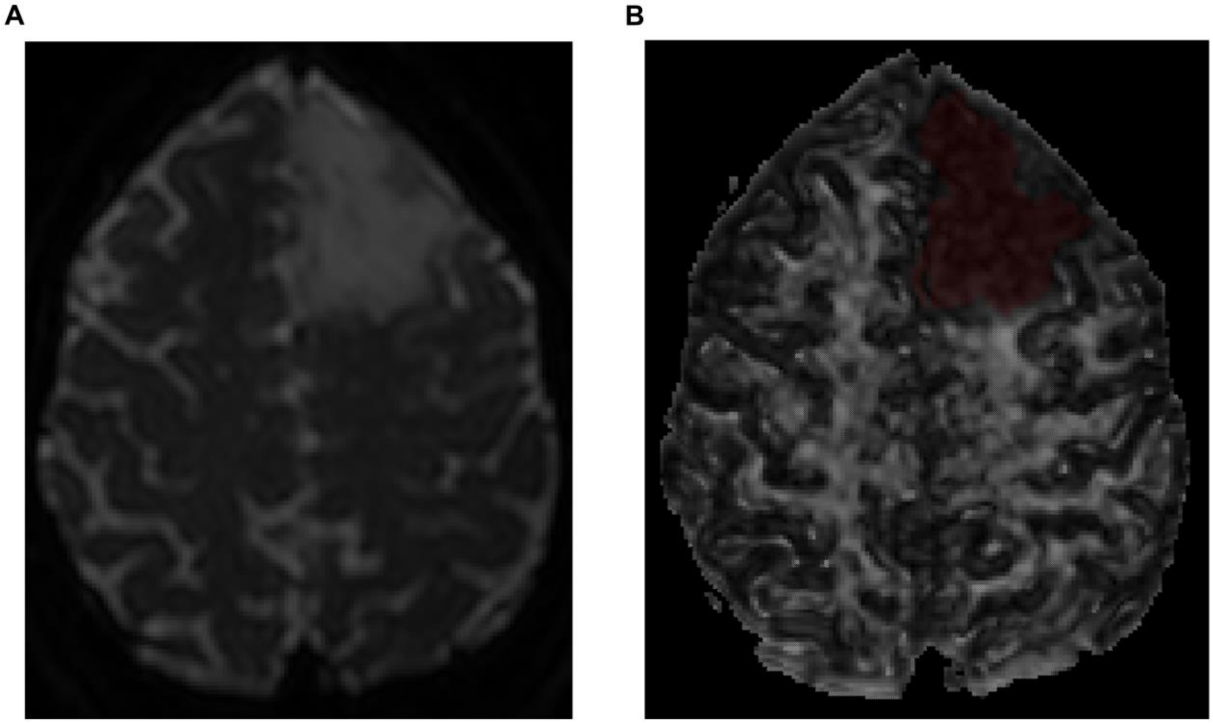

Supplementary Table 1

Feature	Garson importance
Voxel.Count	0.052879542
FA.LBP.23	0.044340319
B0.LBP.15	0.02869439
B0.LBP.31	0.027547611
FA.LBP.13	0.024239866
B0.LBP.16	0.020719686
B0.LBP.42	0.019661059
FA.LBP.19	0.018245614
FA.LBP.12	0.017457752
FA.LBP.29	0.017309917
B0.LBP.3	0.015644399
B0.LBP.33	0.01558524
B0.LBP.37	0.015553271
FA.LBP.9	0.014985789
FA.LBP.16	0.014572472
B0.LBP.14	0.014349946
B0.LBP.11	0.014271484
B0.LBP.44	0.013503774
B0.LBP.29	0.012826077
FA.LBP.32	0.012625382
B0.LBP.5	0.012442007
B0.LBP.21	0.01229929
B0.LBP.19	0.012242083
FA.LBP.49	0.01184872
FA.LBP.2	0.011802826
FA.LBP.18	0.011505641
B0.LBP.35	0.011329024
FA.LBP.24	0.011117831
FA.LBP.26	0.01108304
FA.LBP.50	0.01085861
B0.LBP.4	0.010657736
FA.LBP.33	0.010520196
FA.LBP.38	0.010244123
FA.LBP.6	0.010097233
FA.LBP.31	0.010079488
B0.LBP.50	0.009807063
B0.LBP.41	0.009764029
FA.LBP.46	0.009252392
B0.LBP.25	0.009231191
FA.LBP.15	0.009130369
B0.LBP.23	0.008914933
FA.LBP.14	0.008835054
B0.LBP.39	0.008676845
B0.LBP.10	0.008606998

FA.LBP.48	0.008417627
B0.LBP.7	0.008339434
B0.LBP.20	0.008254443
B0.LBP.18	0.008249018
B0.LBP.17	0.008229843
B0.LBP.30	0.008144929
B0.LBP.6	0.008133031
FA.LBP.11	0.007917632
FA.LBP.21	0.007725761
FA.LBP.4	0.007687854
B0.LBP.49	0.00766622
B0.LBP.48	0.007477107
B0.LBP.13	0.007459151
B0.LBP.9	0.007321062
B0.LBP.27	0.00718996
FA.LBP.8	0.007011252
FA.LBP.22	0.006938885
B0.LBP.32	0.006887869
B0.LBP.40	0.006867737
FA.LBP.5	0.00675889
B0.LBP.28	0.006654987
FA.LBP.20	0.006588076
FA.LBP.36	0.006531903
B0.LBP.45	0.006294442
FA.LBP.10	0.006215457
B0.LBP.24	0.006113809
FA.LBP.45	0.005882284
FA.LBP.41	0.005880423
FA.LBP.42	0.005719061
FA.LBP.30	0.005659682
B0.LBP.36	0.005541907
B0.LBP.43	0.005512425
FA.LBP.17	0.005499567
B0.LBP.2	0.005452586
FA.LBP.47	0.005351752
FA.LBP.3	0.00522506
FA.LBP.1	0.005120527
FA.LBP.43	0.005086328
B0.LBP.38	0.005002051
FA.LBP.44	0.004963411
FA.LBP.28	0.004915192
B0.LBP.1	0.004861277
B0.LBP.22	0.004800087
B0.LBP.12	0.004710828
B0.LBP.26	0.004648606
B0.LBP.34	0.004569496
FA.LBP.27	0.004562085

FA.LBP.34	0.004299266
FA.LBP.39	0.00415498
FA.LBP.7	0.004074589
B0.LBP.8	0.003945895
FA.LBP.37	0.003896323
FA.LBP.40	0.003832376
B0.LBP.47	0.003779355
B0.LBP.46	0.003510904
FA.LBP.25	0.002132345
FA.LBP.35	0.000972642
